# 

**Published:** 1973-07

**Authors:** 


					3,/^5o?3
BRISTOL
MEDICO-
CHIRURGICAL
JOURNAL
if nation JL \
7    \
\
JULY 1973
VOL. 88 (iii) No. 327

				

